# A semiparametric modeling framework for potential biomarker discovery and the development of metabonomic profiles

**DOI:** 10.1186/1471-2105-9-38

**Published:** 2008-01-23

**Authors:** Samiran Ghosh, David F Grant, Dipak K Dey, Dennis W Hill

**Affiliations:** 1 Department of Mathematical Sciences, Indiana University Purdue University, Indianapolis, Indiana, USA; 2 Department of Pharmaceutical Sciences, University of Connecticut, Storrs, Connecticut, USA; 3 Department of Statistics, University of Connecticut, Storrs, Connecticut, USA

## Abstract

**Background:**

The discovery of biomarkers is an important step towards the development of criteria for early diagnosis of disease status. Recently electrospray ionization (ESI) and matrix assisted laser desorption (MALDI) time-of-flight (TOF) mass spectrometry have been used to identify biomarkers both in proteomics and metabonomics studies. Data sets generated from such studies are generally very large in size and thus require the use of sophisticated statistical techniques to glean useful information. Most recent attempts to process these types of data model each compound's intensity either discretely by positional (mass to charge ratio) clustering or through each compounds' own intensity distribution. Traditionally data processing steps such as noise removal, background elimination and m/z alignment, are generally carried out separately resulting in unsatisfactory propagation of signals in the final model.

**Results:**

In the present study a novel semi-parametric approach has been developed to distinguish urinary metabolic profiles in a group of traumatic patients from those of a control group consisting of normal individuals. Data sets obtained from the replicates of a single subject were used to develop a functional profile through Dirichlet mixture of beta distribution. This functional profile is flexible enough to accommodate variability of the instrument and the inherent variability of each individual, thus simultaneously addressing different sources of systematic error. To address instrument variability, all data sets were analyzed in replicate, an important issue ignored by most studies in the past. Different model comparisons were performed to select the best model for each subject. The m/z values in the window of the irregular pattern are then further recommended for possible biomarker discovery.

**Conclusion:**

To the best of our knowledge this is the very first attempt to model the physical process behind the time-of flight mass spectrometry. Most of the state of the art techniques does not take these physical principles in consideration while modeling such data. The proposed modeling process will apply as long as the basic physical principle presented in this paper is valid. Notably we have confined our present work mostly within the modeling aspect. Nevertheless clinical validation of our recommended list of potential biomarkers will be required. Hence, we have termed our modeling approach as a "framework" for further work.

## Background

**Figure 1:**
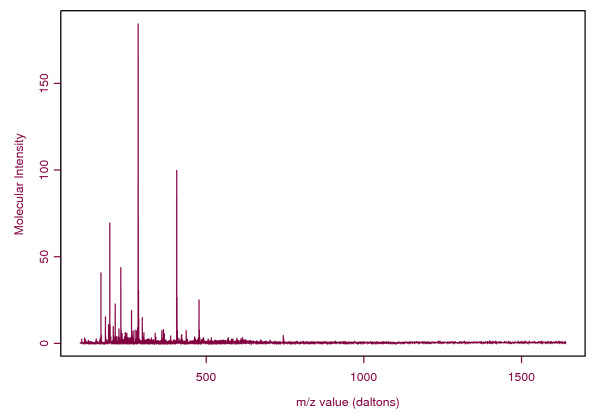
A sample ES/TOF MS spectrum.

Mass spectrometry is an analytical technique for identifying compounds based on their mass to charge (m/z) ratio. It can also be used to quantify the amount of a compound in that the abundance of ions at a given m/z is proportional to the amount of the correlative compound present. With recent advances in this technology a new direction in bioinformatics has emerged for the identification of biomarker patterns that can be used for diagnosis, prognosis or monitoring disease status. The underlying hypothesis is that the mass spectral profile of patients will differ significantly from that of healthy individuals (controls). As might be expected, the methods used for assessing "*significant difference*" vary widely. Starting from different clustering algorithms [1], wavelet based feature extraction [2-5] and other methods have been suggested to understand this "*significant difference*". However none of these models the physical process that generated the data. Several other problems [6] regarding instrument sensitivity and reproducibility of the data set underlying these approaches remain unresolved. A major concern expressed in the literature [7] is the calibration of the mass spectrometer. The data generated by mass spectrometry of urine, blood or serum is composed of the ions of compounds or fragments of compounds identified by their mass-to-charge ratio (m/z) and the abundance of each ion as depicted in Figure 1. Due to inherent analytical variability the same ions in replicates of a sample will have slightly different measured m/z values. Different binning/alignment [7,8] algorithms and clustering techniques [1] have been suggested to properly correlate ions between samples, prior to analysis. While all of these methods are ad hoc it is of interest to model the data with instrument variability as part of the model building process itself. In the present study we address the feasibility of using high resolution mass spectrometry to identify and quantify metabolites as potential biomarkers in the urine of acute trauma patients compared with control urine samples. We postulate that not only are we likely to find specific metabolites that could be used as prognostic indicators of patient outcome, we could also begin to understand the mechanisms responsible for the development of disease progression and outcome in acute trauma patients.

The enormous amount of data generated by mass spectrometric analysis requires sophisticated statistical techniques to differentiate between the urinary metabolic profile of traumatized individuals and healthy individuals. A typical mass spectrum contains thousands of points (m/z values) while subject numbers are quite low. We propose a semiparametric framework to model individual mass spectra obtained from each subject over all replicates. The model or functional form thus developed is termed as subject profile. We have explored different model validation criteria in this regard. Mass spectral profiles of the combined control group and individual patients are compared through a survival relative intensity function (SRIF). A deviation or irregularity in the pattern of the patients from that of the control group indicates possible effects due to trauma. Aided with this knowledge, a mechanism based on predictive relative abundance is recommended to identify potential biomarkers associated with trauma. Though we have shown efficacy of our method for metabonomics this same approach could be easily extended for other domains, as long as the physical mechanism that generates data remains the same.

The rest of the paper is organized as follows. We first develop the semiparametric framework in the Methods section. Choice of prior and development of posterior sampling schemes are discussed next. Following that we describe predictive relative abundance criteria and construction of SRIF for biomarker discovery framework. Then we illustrates different model validation and comparison criteria. We tested our proposed methodology with actual data in the Results and Discussions section. We then provide a brief discussion and direction for future work.

## Methods

### 0.1 Semiparametric Framework

We begin with a raw mass spectrometer data set. Some of the very low intensity ions are removed to eliminate possible noise. Details are instrument specific and hence discussion on this is postponed for the time being and will be revisited in subsection 0.5. Suppose our data came from *N *subjects, where each subject could be either traumatic or control. Data for a single subject will have *r *replicates. Let *D*^*i *^denotes a *n*_*i *_× 2 dimensional spectrum for the i-th replicate, where *i *= 1, 2, ..., *r*. For each spectrum $D^{i}(t)=[d_{t},{\left\{t\right\}}_{t_{1_{i}}}^{t_{n_{i}}}]$ will have two components. First component *d*_*t *_indicates observed relative abundance/intensity at an associated position *t*, for $t=t_{1_{i}},t_{2_{i}},...,t_{n_{i}}$. Due to the inherent variability of the mass-spectrometer, intensities may get registered at slightly different positions even within the replicates of a single subject. For the computational stability associated with Markov chain Monte Carlo (MCMC) described latter on, we have log-transformed all the observed m/z values. In the present context *T *denotes the time-of-flight for the particles detected in a mass spectrometer. Since a monotone function converts time-of-flight *T *to an *m/z *value, they have a one to one correspondence and we would not differentiate between them in our future analysis. We can further assume that *f*(*t*) denotes the probability density function of the stochastic process underlying *T*. The *h*(*t*) termed as intensity function, which denotes the instantaneous rate of ions hitting the detector at time *t*. This in turn denotes instantaneous rate of change in the density function *f*(*t*), i.e., $h(t)=\lim_{\Delta t\to 0}\frac{F(t+\Delta t)-F(t)}{\Delta t(1-F(t))}$ which is identical to the hazard function we observe in survival analysis literature. If *h*(*t*) is high, it indicates hitting rate or detection rate is also high. In particular *h*(*t*)Δ*t *is the approximate probability of hitting the detector in (*t*, *t *+ Δ*t*], given that the ion has not arrived at the detector (hence not detected) up to time *t*. We will not differentiate between the event of arrival of the ions at the detector of the mass spectrometer and the actual detection (current flow). In reality there is a very tiny time lag between these two events. The function *h*(*t*) has two desirable properties

$$\begin{array}{ccc} h(t)\geq 0 & \text{and} & \int_{0}^{\infty }h(t)dt=\infty . \end{array}$$

Moreover at any instance *t*, the area under the curve *h*_*i *_(*t*) is given by $H(t)=\int_{0}^{t}h(u)du$. It is possible to calculate the approximate value of *H *(*t*) from any given spectrum. For modeling *H *(*t*) we will use the representation theorem for monotone functions [9] on [0, 1]. However note that *H *: ℝ^+ ^→ ℝ^+^. To convert its range in the [0, 1], we would consider the transformation

$$H^{*}(t)=\frac{H(t)}{H(t)+1}.$$

Note that H* : ℝ^+ ^→ [0, 1]. Having transformed the mean intensity into the interval [0, 1] we would like to approximate $H^{*}(t)=\sum_{l=1}^{m}\eta_{l}IB(\tilde{H}(t);r_{l},s_{l})$, where *η*_*l *_are weights satisfying $\sum_{l=1}^{m}\eta_{l}=1$ for all replicates and *I B*(.; *r*_*l*_, *s*_*l*_) denotes the incomplete beta function. Collecting the weights into ***η ***= (*η*_1_, *η*_2_, ... *η*_*m*_) a Dirichlet prior can be assigned. The (*r*_*l*_, *s*_*l*_) are chosen to ensure that Beta c.d.f's have equally spaced means and are centered around $\tilde{H}$(*t*). $\tilde{H}$(*t*) is a suitable function which maps the time scale into [0,1]. The related issue of choosing $\tilde{H}$(*t*) need to be resolved by selecting a plausible central function around which the H* function is distributed. If we consider cumulative intensity function, parallel to the cumulative hazard function of the survival analysis literature [10] then we can take $\tilde{H}(t)=\frac{{\tilde{H}}_{0}(t)}{1+{\tilde{H}}_{0}(t)}$. We would like to use Extreme value, Double exponential and Normal distribution for $\tilde{H}$_0_(*t*) and perform model comparison. We consider different choices of $\tilde{H}$_0_(*t*) as

• Extreme value : $f_{0}(t)=\frac{1}{\beta }e^{\frac{t-\alpha }{\beta }}\exp(-e^{\frac{t-\alpha }{\beta }})$, ${\tilde{H}}_{0}(t)=e^{\frac{t-\alpha }{\beta }}$; *γ *= (*α*, *β*) and *β *> 0,

• Double Exponential : $f_{0}(t)=\frac{1}{2\beta }e^{-\left|\frac{t-\alpha }{\beta }\right|}$, ${\tilde{H}}_{0}(t)=\{\begin{array}{ll} -\log\left[1-\frac{1}{2}\exp\left(\frac{t-\alpha }{\beta }\right)\right], & t\leq \alpha \\ \frac{t-\alpha }{\beta }+\log2, & t\geq \alpha \end{array};\gamma =(\alpha ,\beta )$ and *β *> 0,

• Normal : $f_{0}(t)=\frac{1}{\sigma \sqrt{2\pi }}\exp\left\{-\frac{1}{2}{\left(\frac{t-\mu }{\sigma }\right)}^{2}\right\}$, ${\tilde{H}}_{0}(t)=-\log\left\{1-\Phi \left(\frac{t-\mu }{\sigma }\right)\right\}$; *γ *= (*μ σ*), *σ *> 0.

The $\tilde{H}$_0_(*t*) is serving the role of a central function and a flexible choice of this will enable us to capture non-standard patterns in the profile in a microscopic sense. Note that

(1) $$\begin{array}{l} H^{*}(t)=\frac{H(t)}{H(t)+1}\Rightarrow H(t)=\frac{H^{*}(t)}{1-H^{*}(t)}=\frac{\sum_{l=1}^{m}\eta_{l}IB(\tilde{H}(t);r_{l},s_{l})}{\left\{1-\sum_{l=1}^{m}\eta_{l}IB(\tilde{H}(t);r_{l},s_{l})\right\}},\\ h(t)=\frac{d}{dt}H(t)=\frac{d}{dt}\left(\frac{H^{*}(t)}{1-H^{*}(t)}\right)=\frac{\frac{d}{dt}\tilde{H}(t)\sum_{l=1}^{m}\eta_{l}Be(\tilde{H}(t);r_{l},s_{l})}{{\left\{1-\sum_{l=1}^{m}\eta_{l}IB(\tilde{H}(t);r_{l},s_{l})\right\}}^{2}}, \end{array}$$

where $\frac{d}{dt}\tilde{H}(t)=\frac{\frac{d}{dt}{\tilde{H}}_{0}(t)}{{\left(1+{\tilde{H}}_{0}(t)\right)}^{2}}$ Straight forward calculation parallel to survival analysis literature shows that

(2) $$f(t)=h(t)\exp(-H(t))=\frac{\frac{d}{dt}\tilde{H}(t)\sum_{l=1}^{m}\eta_{l}Be(\tilde{H}(t);r_{l},s_{l})}{{\left\{1-\sum_{l=1}^{m}\eta_{l}IB(\tilde{H}(t);r_{l},s_{l})\right\}}^{2}}\exp\left(-\frac{\sum_{l=1}^{m}\eta_{l}IB(\tilde{H}(t);r_{l},s_{l})}{1-\sum_{l=1}^{m}\eta_{l}IB(\tilde{H}(t);r_{l},s_{l})}\right).$$

### 0.2 Likelihood Construction

If we have *n *many distinct intensity values detected by the detector and *t*_*j *_denotes the observed time of flight (TOF) for the j-th (*j *= 1, ..., *n*) batch of *d*_*j *_many ions, then the likelihood is

$$L=\prod_{j=1}^{n}\{f(t_{j})){\}}^{d_{j}}=\prod_{j=1}^{n}{\left[h(t_{j})\exp\{-H(t_{j})\}\right]}^{d_{j}}.$$

In the present context we do not know the exact number of ions, rather we will observe *d*_*t *_the relative intensity or relative amount of current flow detected by the mass spectrometer at *t*-th m/z value. However considering the idealized experiment, the amount of current flow is proportional to the number of ions detected at any associated position and hence used here as a proxy. The observed m/z value (*t*) will have time stamp at $t_{1_{i}},t_{2_{i}},...,t_{n_{i}}$, for the i-th replicate. The complete data likelihood for a subject having *r *replicates can be expressed as

(3) $$\begin{array}{lll} L(\eta ,\gamma ) & = & \prod_{i=1}^{r}\prod_{t=t_{1_{i}}}^{t_{n_{i}}}{\left[h(t)\exp\{-H(t)\}\right]}^{d_{t}}\\ & = & \prod_{i=1}^{r}\prod_{t=t_{1_{i}}}^{t_{n_{i}}}{\left(\frac{\frac{d}{dt}\tilde{H}(t)\sum_{l=1}^{m}\eta_{l}Be(\tilde{H}(t);r_{l},s_{l})}{{\left\{1-\sum_{l=1}^{m}\eta_{l}IB(\tilde{H}(t);r_{l},s_{l})\right\}}^{2}}e^{\left(-\frac{\sum_{l=1}^{m}\eta_{l}IB(\tilde{H}(t);r_{l},s_{l})}{1-\sum_{l=1}^{m}\eta_{l}IB(\tilde{H}(t);r_{l},s_{l})}\right)}\right)}^{d_{t}}. \end{array}$$

An interesting departure from the regular hierarchical model setup is that the above likelihood does not integrate all the subjects at once. The same model is fitted for different subjects by integrating over all subject specific replicates to capture subject specific variations. Note that *γ*, the vector of parameters associated with $\tilde{H}$(*t*) and *f *(*γ*) is a corresponding suitably chosen prior. Clearly, likelihood is a function of mixing weights ***η ***= (*η*_1_, *η*_2_, ... *η*_*m*_). With the above data likelihood specific choice of $\tilde{H}$(*t*) and adjoining suitable priors to the ***η ***and *γ*, a complete Bayesian hierarchical model setup is completed. We further elucidate the choice of different priors and explicit steps for numerical calculation in next section.

## Prior Choices and Numerical Implementation

With the notation of earlier section the joint posterior distribution of the parameters of interest namely Θ = (***η***, *γ*) under suitable prior on ***η ***and *γ *is given by

(4) $$\begin{array}{r} f\left(\eta ,\gamma |{\left\{t\right\}}_{t=t_{1_{i}}}^{t_{n_{i}}},h_{i}(t),i=1,...,r\right)\propto f(\eta |\phi_{\eta })\times f(\gamma )\times \\ \prod_{i=1}^{r}\prod_{t=t_{1_{i}}}^{t_{n_{i}}}{\left(\frac{\frac{d}{dt}\tilde{H}(t)\sum_{l=1}^{m}\eta_{l}Be(\tilde{H}(t);r_{l},s_{l})}{{\left\{1-\sum_{l=1}^{m}\eta_{l}IB(\tilde{H}(t);r_{l},s_{l})\right\}}^{2}}e^{\left(-\frac{\sum_{l=1}^{m}\eta_{l}IB(\tilde{H}(t);r_{l},s_{l})}{1-\sum_{l=1}^{m}\eta_{l}IB(\tilde{H}(t);r_{l},s_{l})}\right)}\right)}^{d_{t}}. \end{array}$$

The flexibility of our approach comes from modeling the ***η***, which gives subject-specific information. It is natural to model ***η ***as *f *(***η***|*φ*_*η*_), is the Dirichlet prior drawn from *Dirichlet *(*φ*_*η *_**1**), where we take a fixed scalar hyper-parameter $\phi_{\eta }=\frac{1}{m}$, as in our case *m *= 5. As pointed out by [10] this choice combined with evenly spaced beta distribution parameter, namely *r*_*l *_and *s*_*l *_will give rise to a model centered around $\tilde{H}$(*t*).

We fix *m *= 5, {*r*_*l*_} = (1, 2, 3, 4, 5) and {*s*_*l*_} = (5, 4, 3, 2, 1) which will produce five evenly spaced beta distributions. Five beta mixtures should not be mixed with the coarseness of the MS data. It should be noted that the present modeling effort is concentrated on the *hazard *space where five beta mixtures are used as an artifact with different choice of centering functions to capture wide range of intensity functions. A flexibility analysis of the presented model is also included in the supplementary material (see Additional file 1). Parameters associated with *γ *are either location or scale parameters. For the location parameter (*α *or *μ*) we have chosen a Gaussian prior with sufficiently large variance. Since the observed relative intensity even in the log scale does not go below 2 (minimum resolution is 100 daltons in the mass spectrometer), some truncation is necessary to achieve this goal. We have used truncated normal distribution (truncated any value that goes below 0). From a practical point of view this decision makes MCMC sampling scheme to converge much faster while used in conjunction with normal proposal. For scale parameter (*β *or *σ*^2^) initially an inverse gamma prior is assigned. Though this works well for Extreme value distribution, for other two cases we found that MCMC chain is very slow from jumping one state to another. Thus for those cases, a log transformation is used for the scale parameter which also helps to symmetrize the posterior distribution. A normal proposal is used for this transformed case. Nevertheless the posterior distribution is always complex and implementation of this Bayesian procedure requires MCMC sampling scheme. In general it is difficult to show log-concavity of the conditional posterior distribution of ***η ***in its each component. Keeping this in mind and for the computational flexibility we would make a transformation for ***η***. Since the support of each *η*_*l *_is [0, 1] we make change of variable for (*η*_1_, *η*_2_, *η*_3_, *η*_4_) to (*θ*_1_, *θ*_2_, *θ*_3_, *θ*_4_), where $\theta_{l}=\log\left(\frac{\eta_{l}}{1-\eta_{l}}\right)$ for *l *= 1, ..., 4. Note that we need to specify only four of the *η*_*l*_'s as $\eta_{5}=1-\sum_{l=1}^{4}\eta_{l}$. The inverse transformation is $\eta_{l}=\frac{\exp(\theta_{l})}{1+\exp(\theta_{l})}$, with Jacobian $\frac{\exp(\theta_{l})}{{\left[1+\exp(\theta_{l})\right]}^{2}}$. The above transformation makes it easier to specify the proposal density, although one can construct Metropolis chain for the untransformed parameter. The proposal distribution is normal $N_{4}(\theta_{l}^{\left(t-1\right)},\hat{\Sigma })$, where initial estimates are obtained through Nelder-Mead downhill simplex algorithm. To accelerate convergence from the output of the first algorithm we got a new estimate of $\hat{\Sigma }$ as $\frac{1}{G}\sum_{g=1}^{G}\left(\theta_{j}-\bar{\theta })(\theta_{j}-\bar{\theta }\right)$, where *g *indexes Monte Carlo samples. The sampling scheme implemented is a variation of the grouped Gibbs sampler [11,12], which requires drawing:

1. ***θ ***~ *π *(***θ***|*γ*, $D=\cup_{i=1}^{r}D^{i}$), with acceptance probability $\alpha =\min\left\{1,\frac{\pi (\theta |\gamma ,D)N_{4}(\theta_{0},\hat{\Sigma })}{\pi (\theta_{0}|\gamma ,D)N_{4}(\theta ,\hat{\Sigma })}\right\}$ and then transform back ***θ ***↦ ***η***,

2. *μ *~ *π *(*μ*|***η***, *σ p*, *D*),

3. *σ *~ *π *(*μ*|***η***, *μ*, *p*, *D*).

Since full conditionals are not in conventional form we approximate them by sampling with a Metropolis step within the Gibbs sampler. Under this setup ***η ***and *γ *are updated sequentially and monitored for convergence following post burn-in period. We would like to mention at this point that log-transformation of the observed m/z values are done for greater numerical stability of the MCMC algorithm only.

## Predictive Calculation

We have a two folded objective namely subject diagnostics and potential biomarker identification. Notably subject diagnostic is a global summarization while biomarker (m/z values) identifications are more localized in nature. For this we proposed two criteria namely Survival Relative Intensity Function (SRIF) and Predictive Relative Abundance (PRA). SRIF is calculated at low resolution, the very essence being global estimation of subject profile (a representative curve describing overall rough shape) for general subject diagnostic. PRA is directed to identify potential biomarkers associated with anomalous m/z values at much finer resolution. This two step breakdown of estimation at different resolution is essential for successful information retrieval from highly irregular MS data.

### 0.3 Predictive Relative Abundance

For prediction purposes we would like to introduce a few more notations. Let Ni(=∑t=t1itnidt) denote the total relative intensity observed at the i-th replicate. $n_{t}^{i}$ is the number of ions at risk of hitting the detector at a time just prior to time *t *in the i-th replicate. We can easily calculate nti=Ni−∑t=t1itni−1dt. True relative abundance at the *t*-th m/z value for the i-th replicate is denoted as $\delta_{t}^{i}$. An estimate of $\delta_{t}^{i}$ termed as predictive relative abundance can be found through

$${\hat{\delta }}_{t}^{i}=\hat{h(t)}\times n_{t}^{i}\text{ for }t=t_{1_{i}},t_{2_{i}},...,t_{n_{i}}.$$

A good estimate should have small difference between ${\hat{\delta }}_{t}^{i}$ and its observable counterpart *d*_*t*_. A mean square based criterion is being provided in the supplementary material (see Additional file 1), exploiting this difference for model fitting purpose. In case we have drawn *G *many MCMC samples then an average MC estimate will be

$${\hat{\delta }}_{t}^{i}=n_{t}^{i}\times \frac{1}{G}\sum_{g=1}^{G}\hat{h_{g}(t)}.$$

To get an estimated value of *h*(*t*) through equation (1) the only requirement is the differentiability of $\tilde{H}$(*t*), which in turn depends upon the differentiability of $\tilde{H}$_0_(*t*). Hence once the parameter in Θ is estimated through MCMC, we can easily calculate $\hat{h(t)}$. Under the assumption that control group is homogeneous, posterior distribution for each model parameter should have similar distribution across all controls. To illustrate more, consider a single parameter only (say *α*). We obtain posterior samples from all control subjects separately and then mix them together under the hypothesis that posterior distribution of *α *is same for all controls. This gives us resultant posterior distribution combining all controls for *α*. This pooled sample is then used to construct ${\hat{\delta }}_{t}^{i}$, and its 95% credible interval. This will give us a upper and lower bound within which an observed relative abundance (*d*_*t*_) at any m/z value should be lying for any normal subject. We hypothesize that deviation from the above will indicate possible effects of trauma.

### 0.4 Product-Limit type Estimate of SRIF

The probability that an individual ion has not arrived (hence not being detected) until time *t *is given by *S*(*t*)(= *P*(*T *> *t*)). This will be termed as survival relative intensity function (SRIF) or so called profile function. We have entertained a product-limit (Kaplan-Meier type) estimate of *S*(*t*) defined as

(6) $$S(t)=\prod_{j:t_{j}\leq t}\left(1-\frac{d_{t_{j}}}{n_{t_{j}}}\right).$$

For any subject this is an observable quantity. However since we can estimate and more importantly get a confidence bound of the estimate of *d*_*t *_through ${\hat{\delta }}_{t}^{i}$, an estimate of the SRIF for the i-th replicate of a subject will be

$$\hat{S^{i}(t)}=\prod_{j:t_{j}\leq t}\left(1-\frac{{\hat{\delta }}_{t_{j}}^{i}}{n_{t_{j}}^{i}}\right)=\prod_{j:t_{j}\leq t}\left(1-\hat{h(t_{j})}\right).$$

Where *i *is the indicator of the corresponding replicate number. A Monte-Carlo estimate of *S*(*t*) will be

$$\hat{S_{MC}^{i}(t)}=\frac{1}{G}\sum_{g=1}^{G}\hat{S_{g}^{i}(t)}=\frac{1}{G}\sum_{g=1}^{G}\prod_{j:t_{j}\leq t}\left(1-\hat{h_{g}(t_{j})}\right).$$

Since ${\hat{\delta }}_{t}^{i}$ is a function of Θ, so does *S*(*t*). Once the overall pulled estimates of the parameters of Θ based on the control group is obtained, we would like to treat *S*(*t*) thus obtained from the control group as the representative surviving relative intensity function (SRIF) of a normal individual being. Similar to the relative abundance case we would like to draw 95% credible interval for the ideal SRIF obtained from the control group. For a patient, we would like to compare this ideal SRIF band with that of the individually observed SRIF obtained through equation (6).

As mentioned earlier, SRIF will give us an overall visual idea about an individual's well-being. Depending upon this initial health identification status, we will recommend further investigation through predictive relative abundance bound described earlier (subsection 0.3), for identifying abnormal relative abundance associated with specific individual m/z value. Thus identified m/z values are further recommended for compound level identification as potential biomarker associated with traumatic disorder. Some additional discussion regrading the nature and computation of SRIF and PRA is also provided in the supplementary material (see Additional file 1).

## Different Model Comparison Criteria

In the present context we have entertained three different model choices, which necessitates model comparison for getting the best fitted model. We have explored both Bayesian as well as some frequentist model selection criteria. While some of them are readily applicable, some requires modification to be meaningful for mass spectrometry data set. We have used Conditional Predictive Ordinate (CPO), Bayes Factor, Bayesian Information Criterion and a variant of Mean Square Based Criterion for model assessment. To check predictive accuracy, a new measure namely Conditional Predictive Intensity/Hazard Function (CPIF) has been developed. We have also discussed in details different estimation strategy involving Monte Carlo approximation of the above measures. For the sake of space statistical justification and calculation behind all of these criteria are placed in the supplementary material (see Additional file 1). We would like to note that DIC is not being explored in this paper as it is under severe scrutiny for its lack of interpretation in different scenario. However its implementation is not difficult and can be easily done if required.

## Results and Discussion

### 0.5 Data Description: Trauma Data Set

Acute trauma with associated hemorrhage, shock and sepsis, often produces a generalized inflammatory response resulting in organ dysfunction and organ failure. Acute trauma is associated with high mortality rates and is among the leading causes of death in Americans between the ages of 1 and 44 [13]. In this paper we explore the feasibility of using a high resolution mass spectrometry to identify and quantify metabolites in the urine of acute trauma patients and compare these with control urine samples. The rationale behind this approach is that such data would provide a "metabonomic profile" that could be used for real-time analysis of acute trauma patient status. It should be noted that trauma is not a disease and is very much heterogenous in nature. Each trauma patient likely to be unique. This points out that though normal subjects can be combined to develop a standard "metabonomic profile", patients may not necessarily be combined unless we can create some homogenous traumatic disorder in laboratory controlled environment. Our goal is to determine whether a patient profile is anomalous in comparison to standard metabonomic profile and then detect patient specific signals for individual trauma patients. With these goals in mind we have retrospectively analyzed urine samples collected from 6 normal healthy individuals and 6 patients with different degree of acute trauma. Each urine sample was analyzed in triplicate (*r *= 3). Every patient is coded as "P-DG", while for every control it is "C-DG". Creatinine concentrations were measured [14] in each urine sample and each sample was analyzed by electrospray ionization on a Micromass Q-TOF2 mass spectrometer using lisinopril as a calibration and quantitative internal standard. Mass spectra were collected in positive ionization mode from 100 to 1600 daltons. Mass spectral data was collected in the continuum mode and processed by MassLynx (Micromass) software to generate centroid spectral data consisting of accurate m/z values and ion intensities. The data used in the present study were generated by eliminating all ions that were less than or equal to three times the instrument noise and dividing the intensity of each ion by the concentration of creatinine in the respective urine sample. The normalization of the ion intensities to the creatinine concentration was necessary to compensate for urine dilution in different individuals. Ion resolution in this system was between 9200 and 9800 (Δ*m*_1/2_/*m*) and m/z reproducibility was less than +/- 20 ppm. The m/z derived from a specific compound may therefore vary by the reproducibility of the method if the ion is resolved from the ions produced by other compounds. As described in section 0.1, we have entertained three different model choices; Model 1: $\tilde{H}$_0_(*t*) corresponds to Extreme value, Model 2: $\tilde{H}$_0_(*t*) corresponds to Double Exponential and Model 3: $\tilde{H}$_0_(*t*) corresponds to Normal distribution. All of these models have seven parameters, which need to be estimated through MCMC. The first 5,000 iterations were thrown out as burn-in period, then an additional 50,000 iterations were obtained out of which we accepted only every 50-th iteration, creating 1000 samples from the posterior distribution. This was used to compute all necessary statistics namely posterior mean, 95% credible interval and other measure related to model comparison. Every MCMC chains were tested for convergence by Geweke's [15] convergence diagnostic and autocorrelation plot through R software. Several combinations of hyperparameter values were tried. We report the results for prior distribution of the location parameter as *N *(2, 10) and for scale parameter as *IG *(2, 10), so that priors do not drive the inference.

### 0.6 Model Comparison Results

Different model parameter estimates, standard deviation and their 95% highest posterior density (HPD) credible intervals are placed in the supplementary material (see Additional file 1) and can easily accessed by interested reader. We performed rigorous model selection on the basis of different criteria described earlier. On the basis of log of Pseudo Marginal Likelihood, Bayes factor and BIC, clearly Model 2, based upon Double Exponential distribution is outperforming other models. For some situations Model 1 was doing better. Notably for MSE and cross validation based approach Model 1 was doing better and Model 2 was close second. Model 3 based on normal distribution is not performing well. To save space all model selection results are placed in the supplementary material (see Additional file 1). Though the results are not reported here, we have also tried a mixture of two normal distribution, but this choice produces no improvement. We acknowledge that the decision of choosing the best model is subjective, especially considering so many different model selection criteria all together and in fact there may not be any unique and unanimous model choice in the real world. However, combining all the criterion we suggest the Double Exponential based model as being best model choice for subject diagnostic and bio-marker detection purpose. Notably Model 1 is also a strong contender and other mixture model based approach may be taken for modeling $\tilde{H}$_0_(*t*). However we would like to investigate them else where in future.

### 0.7 Subject Diagnostic and Mechanism for Biomarker Identification

**Figure 2:**
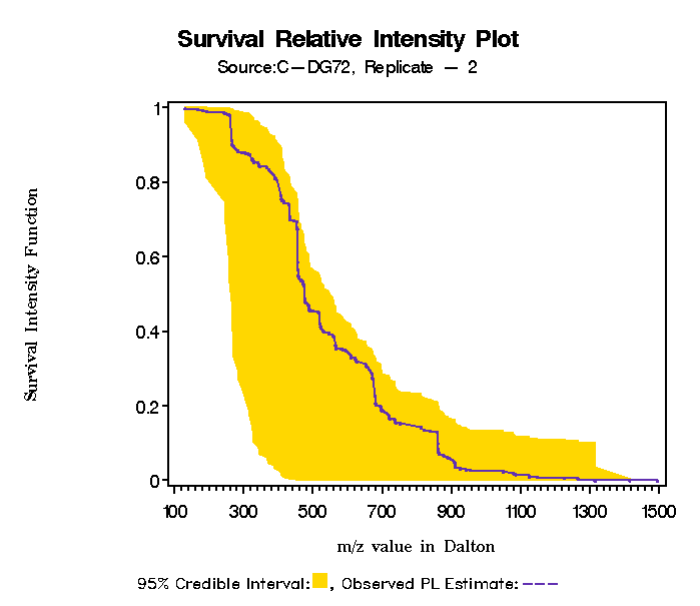
Survival Relative Intensity Function plot for control C-DG72. The shaded region denotes 95% credible interval. Note that subject C-DG72 is not being used in this particular credible interval construction hence it supports cross validation. Ideally for normal individual we will expect the SRIF to be completely inside the credible interval constructed from remaining normal subjects.

**Figure 3:**
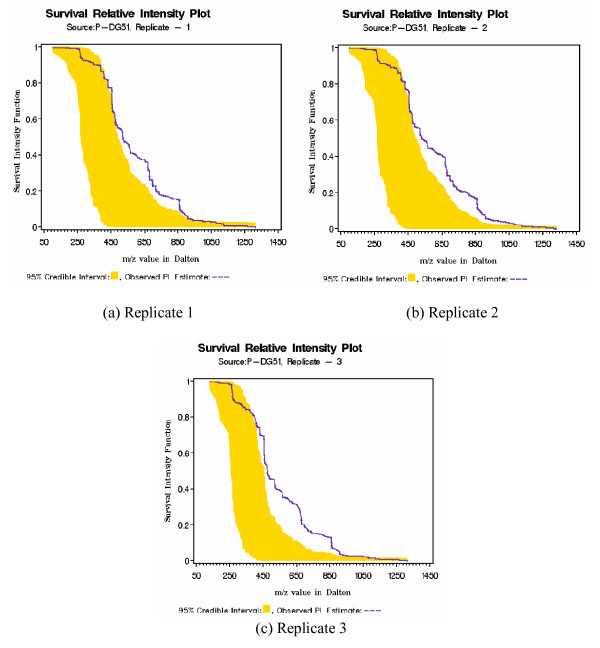
Survival Relative Intensity Function plot for patient P-DG51. Large deviation from the 95% confidence band is an indication of irregular metabolic behavior and thus needs further investigation.

Once we have selected a model we have a two goals. Through the SRIF function we can determine whether the observed subject SRIF deviates outside the 95% credible interval constructed from controls. The underlying hypothesis is that for a normal subject the observed product limit estimate of SRIF remains within the credible interval, while for patients it will deviate outside the credible interval. An obvious advantage of our semiparametric Bayesian modeling approach is that we are producing 95% credible interval which will not be present in traditional Kaplan-Mier type estimate. In Figure 2 we have plotted SRIF for the control C-DG72. Since the observed SRIF lies completely inside the 95% credible interval, this gives an ideal picture we should expect for a normal individual. Note that control individuals are just normal human being, and that their behavior is far from the realm of laboratory controlled experimental environment. For that reason small degree of deviation in the SRIF even among the controls may be observed and expected due to inherent and unavoidable biological variation. However among the patients the degree of deviation in SRIF is much higher. An example of this is depicted in Figure 3 for patient P-DG51. Similar results are obtained for other patients (see Additional file 1). Notable 95% credible interval for SRIF is not same across all figures. This is due to the fact that observed intensities were registered at different m/z values for all samples (even among replicates) and credible interval is only constructed at those registered values given the sample. More explanation on this is also provided in the supplementary material (see Additional file 1).

**Figure 4:**
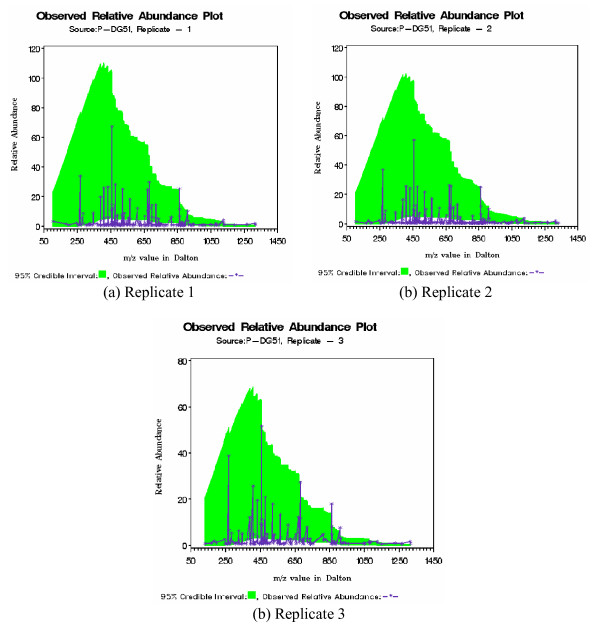
Predictive Relative Abundance plot for patient P-DG51. Those m/z values falling outside (either too large or too small) of 95% credible interval are potential biomarkers for traumatic disorder.

**Figure 5:**
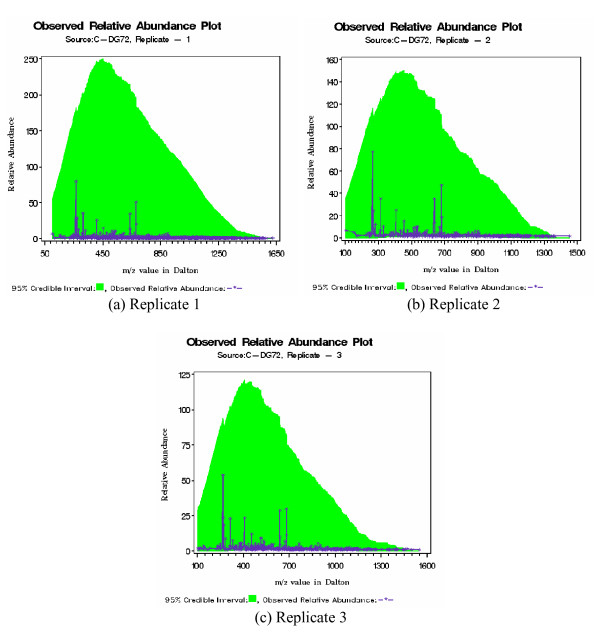
Predictive Relative Abundance plot for control C-DG72. Note that credible interval are constructed using only other five controls. None of the m/z values are outside credible band, which is quite expected for controls.

Next we have plotted predictive relative abundance for patient P-DG51 (Figure 4). The shaded area denotes 95% credible interval drawn as outlined in section 0.3. For all replicates there are several m/z values that are falling outside this shaded region. Our goal is to collect these outlying m/z values and recommend them for further chemical identification as potential biomarkers. Similar conclusion could be drawn for other patients (can be found in the Additional file 1). As a final example we have drawn similar figures for control C-DG72. We have obtained 95% credible interval through remaining five controls and all of the m/z values are inside this interval (Figure 5).

## Conclusion

In this paper we have demonstrated a novel semiparametric modeling technique for analyzing metabonomic data of acute trauma subjects using mass spectrometry. We have proposed modeling the intensity function, using a mixture of incomplete Beta functions. Also rather than traditional point based modeling we have modeled the whole curve or subject profile through this approach. Different model comparison criterion were used for selecting the best model, both from a classical and Bayesian point of view. We have used a mixture of five Betas for our modeling purpose. A more flexible approach will be to use variable number of mixture components, which will necessitate use of the reversible jump MCMC algorithm.

We would also like to emphasize the advantages of modeling a profile. First, spatial correlations that may exist among chemically related m/z values will be automatically incorporated into the model. Secondly, due to inherent instrument variability, specific or static m/z values may loose their true meaning when compared across different subjects. This again questions the traditional point by point comparison of m/z values across different samples. In fact researchers already perceived this problem and for that reason most [1,7,16] mass spectrometry based modeling begins with an alignment or clustering algorithm. Our method is general enough to be applicable with or without alignment. In the absence of any alignment, credible intervals constructed will be wider. This is reasonable considering principles of signal to noise strength. However utmost care should be taken before making any such alignment as wrongly aligned m/z values will result in large false positives, irrespective of the efficacy of latter used methods.

## Authors' contributions

SG is the lead author and conceived the idea of modeling TOF-MS data in survival analysis framework. All computer programming and other derivations are primarily done by him. DFG helped conceive the sampling protocols and design of the study and participated in writing and editing of the manuscript. DD participated in the refinement of the statistical modeling as well in editing the manuscript. DJH helped in the analytical analysis of the samples and helped conceive the sampling protocols and design of the study. All authors have read and approved the final manuscript.

## Supplementary Material

Additional file 1 Additional modeling results and discussions are provided in a separate supplemental file. Click here for file

## References

[B1] Tibshirani R, Hastie T, Balasubramanian N, Scott S, Gongyi S, Albert K, Quynh-Thu L (2004). Sample classification from protein mass spectrometry, by peak probability contrasts. Bioinformatics.

[B2] Timothy WR, Yutaka Y (2004). Multiscale Processing of Mass Spectrometry Data. Tech Rep Working Paper 230, UW Biostatistics Working Paper Series, Fred Hutchinson Cancer Research Center.

[B3] Kwon DW, Tadesse MG, Sha N, Pfeiffer RM, Vannucci M (2006). Identifying Biomarkers from Mass Spectrometry Data with Ordinal Outcome. Cancer Informatics.

[B4] Randolph TW, Tasui Y (2006). Multiscale processing of mass spectrometry data. Biometrics.

[B5] J M, Combes RK, Baggerly K, Kobayasi R (2005). Feature extraction and quantification for mass spectrometry data in biomedical application using the mean spectrum. Bioinformatics.

[B6] Coombes RK, Morris SM, Hu J, Edmonson RS, Baggerly AK (2005). Serum proteomics profiling – a young technology begins to mature. Nature Biotechnology.

[B7] Yutaka Y, McLerran D, Bao-Ling A, Marcy W, Thornquist M, Ziding F (2003). An Automated Peak Identification/Calibration Procedure for High-Dimensional Protein Measures From Mass Spec trometers. Journal of Biomedicine and Biotechnology.

[B8] Kazmi AS, Ghosh S, Shin DG, Hill DW, Grant FD (2006). Alignment of high resolution mass spectra: Development of a heuristic approach for metabolomics. Metabolomics.

[B9] Diaconis P, Ylvisaker D, Bernardo JM, Berger JO, Smith AFM (1985). Quantifying prior opinions. Bayesian Statistics 2.

[B10] Gelfand A, Mallick BK (1995). Bayesian analysis of proportional hazards model built from monotone functions. Biometrics.

[B11] Geman S, Geman D (1987). Stochastic relaxation, Gibbs distribution and the Bayesian restoration of images. IEEE Trans on Pattern Anal and Mach Intel.

[B12] Gelfand AE, Smith AFM (1990). Sampling-based approaches to calculating marginal densities. Journal of the American Statistical Association.

[B13] Anderson RN, Smith BL (2003). Deaths: leading causes for 2001. Natl Vital Stat Rep.

[B14] Greenblatt DJ, Ransil BJ, Harmatz JS, Smith TW, Duhme DW, Koch-Weser J (1976). Variability of 24-hour urinary creatinine excretion by normal subjects.. Journal of Clin Pharmacol.

[B15] Geweke J, Bernardo JM, Berger JO, Dawid AP, Smith AFM (1992). Evaluating the accuracy of sampling-based approaches to calculating posterior moments. Bayesian Statistics 4.

[B16] Ghosh S, Dey D, Hill D, Grant FD (2006). Statistical Approach to Metabonomic Analysis of Rat Urine Following Surgical Trauma. Journal of Chemometrics.

