# Supplementary material for: A semiparametric modeling framework for potential biomarker discovery and the development of metabonomic profiles
Source: BMC Bioinformatics. 2008 Jan 23;9:38. doi: 10.1186/1471-2105-9-38 (PMC2253516; doi:10.1186/1471-2105-9-38)
Supplement: Additional file 1 — Additional modeling results and discussions are provided in a separate supplemental file. [file 1471-2105-9-38-S1.pdf]

Supplementary material for the Biometrics paper “*A Semiparametric Modeling Framework for Potential Bio-Marker Discovery and the Development of Metabonomic Profiles”*

We have presented all the model comparison tables and those figures which are not covered in the main paper in the supplementary section. All model selection criteria and their statistical justification are also described in detail. We hope this additional information will prove useful and increase readability of the main paper considerably.

1. Statistical Description of Different Model Comparison Criteria

1.1 CPO Based Criterion

The CPO statistics is a very useful tool in statistical literature, first defined by Geisser (Geisser, 1980) as a diagnostic measure to detect observations discrepant from a given model. For detailed discussion on this please see (Gelfand, Dey and Chang, 1992) and (Sinha and Dey, 1997). In our situation where we have *r* replicates from a single subject, the whole data set for a subject is denoted as $D = \bigcup_{i=1}^{r} D^i$. The CPO for the k-th m/z value in the i-th replicate is defined as,

$$\begin{aligned} CPO_k^i &= f\left(t_{k_i} \middle| \bigcup_{j=1, j\neq i}^{r} D^j \cup D_{-k}^i\right) \\ &= \int f(t_{k_i}|\Theta, D)\pi(\Theta| \bigcup_{j=1, j\neq i}^{r} D^j \cup D_{-k}^i)d\Theta, \end{aligned} \quad (1)$$

where $D_{-k}^i = \left[d_t, \{t\}_{t_{1_i}, t\neq t_{k_i}}^{t_{n_i}}\right]$ A close expression of equation (1) is not possible to obtain; however a Monte Carlo approximation given by (Gelfand, Dey and Chang, 1992) for the CPO_k^i is,

$$\widehat{CPO}_k^i = \left(\frac{1}{G}\sum_{g=1}^{G}\frac{1}{f(t_{k_i}|\Theta^{(g)})}\right)^{-1}, \quad (2)$$

where $\Theta^{(g)}$ denotes the parameter samples at the *g*-th, *g* = 1, 2,…,*G*, iteration of the Gibbs sampler. A large CPO is indicative of the better fit at that specific point. Pseudo marginal is a useful summary statistic based on the sum of the logarithm of the CPO of the individual observation is defined as

$$B^* = \sum_{i=1}^{r}\sum_{k=t_{1_i}}^{t_{n_i}} \log\left(\widehat{CPO}_k^i\right).$$

Models with greater B^* values will indicate better for the specific subject profile.

1.2 Bayes Factor Based Criterion

The MCMC based approach for calculating Bayes factors was developed by (Chib, 1995), and discussed earlier by (Kass and Raftery, 1995). Given the k-th model, (Chib, 1995), has defined the log of Basic Marginal Likelihood Identity (BMI) as

$$\log(m_k(t)) = \log f_k(t|\Theta_k) + \log\pi(\Theta_k) - \log\hat{\pi}(\Theta_k|D), \quad (3)$$

and the estimated Bayes factor for two rival models indexed by k and k' as,

$$B_{kk'} = \exp\{\log(m_k(t)) - \log(m_{k'}(t))\}.$$

We would like to take importance weighted marginal density estimation (IWMDE) based approach suggested by (Chen, 1994) to estimate $m_k(t)$ directly. Let $\Theta^{(g)}$ denote the parameter samples at the g-th, g = 1, 2,…,G, iteration of the Gibbs sampler from posterior distribution. Then IWMDE yields a consistent estimator for the $\hat{m}_k(t)$ as:

$$\widehat{m_k(t)} = \left(\frac{1}{G} \sum_{g=1}^{G} \frac{w(\Theta^{(g)})}{f(t|\Theta^{(g)})\pi(\Theta^{(g)})} \right)^{-1}, \qquad (4)$$

where $w(\Theta^{(g)})$ is a weighted density function with support $\Omega_w \subset \Omega_{\pi(.|D)}$. A good w should be chosen such that it should have similar shape to the conditional marginal density, which is often unknown. However as pointed out in (Chen, 1994) the rough shape of the conditional marginal distribution can be approximately calculated through MCMC samples drawn from $\pi(\Theta \mid D)$. For our purpose we have chosen a *p*-dimensional multivariate normal $N_p(\mu, \Sigma)$ as a joint importance sampling density for w. We have chosen posterior mean and negative Hessian matrix to evaluate μ and Σ. Once the $B_{kk'}$ has been estimated, if it turns out to be greater than one it suggests that data supports *k*-th model over the k'-th model and vice versa.

1.3 Bayesian Information Criterion

Bayes information criterion is a model comparison criterion based on marginal likelihoods. There are various approximation of this criterion exists in the literature. However we would like to use the approximation achieved by (Schwartz, 1978), which for a model with *p* parameters and *n* sample observation is given by

$$BIC = -2 \log f(t|\hat{\Theta}) + p \log(n).$$

The first term of BIC is just involving the likelihood and the second term is a penalty for model complexity. Model with lower BIC score is preferable. Once calculated we can take the difference between BIC's of the two models to get approximate Bayes factor. We would like to refer (Dudely and Haughton, 1997) for further discussion on BIC.

1.4 Mean Square Error Based Approach

Unlike survival analysis domain, in metabonomics domain, primary interest is in δ_t^i, which in turn depends on the intensity function *h(t)*. This is an estimable quantity for all subjects. The whole profile analysis in the current approach works through modeling *h(t)*. For a specific subject the mean square error (MSE) for *m*-th model is given by

$$MSE(m) = \frac{1}{N} \sum_{i=1}^{r} \sum_{t=t_{1_i}}^{t_{n_i}} (d_t - \widehat{\delta_t^i})^2 = \frac{1}{N} \sum_{i=1}^{r} \sum_{t=t_{1_i}}^{t_{n_i}} (d_t - \widehat{h(t)} \cdot n_t^i)^2, \text{ where } N = \sum_{i=1}^{r} \sum_{t=t_{1_i}}^{t_{n_i}} 1.$$

A model with minimum MSE is chosen to be the best model in mean square sense.

1.5 A Cross Validation Measure

A common cross validation criterion is to leave-one-out estimator obtained by evaluating the model parameters with *k*-th observation removed. Let the k-th m/z value be t_{k_i} and its corresponding relative intensity d_{k_i} is being removed from the i-th replicate of a subject to obtain D^i_{-k} as in the subsection 1.1. The available information is denoted as $D^i_{(k)} = \bigcup_{j=1, j\neq i}^{r} D^j \cup D^i_{-k}$. We define $\hat{h}(t_{k_i} \mid D^i_{(k)})$ as the conditional predictive intensity function (CPIF). However estimation of this quantity poses additional challenge. It is computationally infeasible even for a single subject, to estimate $\hat{h}(t_{k_i} \mid D^i_{(k)})$ for thousands of mass spectrometry data point. However through Monte Carlo sample obtained earlier, combined with numerical integration it is possible to estimate $\hat{h}(t_{k_i} \mid D^i_{(k)})$. It is easy to note that for any m/z value or t following holds

$$h(t|D^i_{(k)}) = \frac{f(t|D^i_{(k)})}{1 - F(t|D^i_{(k)})}.$$

The estimation of $f(t \mid D^i_{(k)})$ is straight forward as discussed in equation (2). To approximately estimate $F(t \mid D^i_{(k)})$ we need to perform an additional numerical integration. For any $t \in [0, \infty)$ we choose k previously available points $t_{0_i} = 0, t_{1_i}, t_{2_i}, \cdots, t_{k_i} = t \in [0, \infty)$. For each $t_{l_i}, (l = 1, \cdots, k)$ we calculate $f(t_{l_i} \mid D^i_{(k)})$ through equation (2). A numerical estimate of $F(t_{l_i} \mid D^i_{(k)})$ is obtained as

$$F(t|D^i_{(k)}) \simeq \sum_{l=1}^{k} \frac{1}{2} \left[f(t_{l_i}|D^i_{(k)}) + f(t_{(l-1)_i}|D^i_{(k)}) \right] \times d_{l_i}, \text{ where } d_{l_i} = t_{l_i} - t_{(l-1)_i}.$$

Above works for any $t = t_{k_i}$. We would like to note at t_{0_i} (= 0) there is no detection of ions, hence $d_t = 0$. Once we obtain $\hat{h}(t \mid D^i_{(k)})$ we can define a MSE based cross validation criterion

$$CV(m) = \frac{1}{N} \sum_{i=1}^{r} \sum_{t=t_{1_i}}^{t_{n_i}} \left(d_t - h_i(\widehat{t|D^i_{(k)}}) \cdot n^i_t \right)^2. \qquad (5)$$

Even each of the $r_{k_i} = \left(d_t - \hat{h}(t \mid D^i_{(k)}) n^i_t \right)$ serves as a measure of model discrepancy at the *t*-th data point for $i = 1, 2, \cdots, r,$ and $t = t_{1_i}, t_{2_i}, \cdots, t_{n_i}$. We have reported root mean square error (RMSE) value for this cross validation measure.

2. Additional SRIF and PRA plots

We provided SRIF plot for patient P-DG51 in the main paper. Figure 1-3 represents SRIF for plots of three additional patients. The main point to note that for patients SRIF curve is considerably outside the HPD credible interval obtained from the control subjects. Interestingly for patient P-DG 31, replicate 1, (Figure 2) the SRIF plot is almost within the credible interval.

However for other replicates a large deviation is observed so that we can comfortably assign patient status. This actually strengthens the necessity of replication in MS experiment.

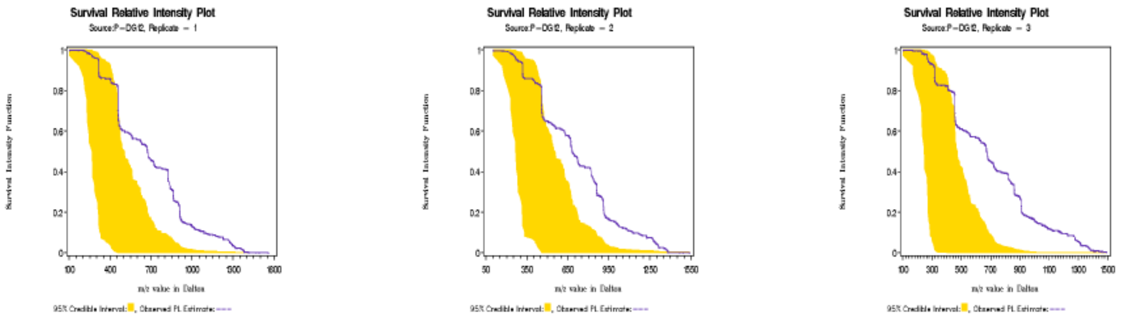

(a) Replicate 1. (b) Replicate 2. (c) Replicate 3.

Figure 1: SRIF plot for patient P-DG12. The large deviation from the 95% confidence band is an indication of irregular metabolic behavior.

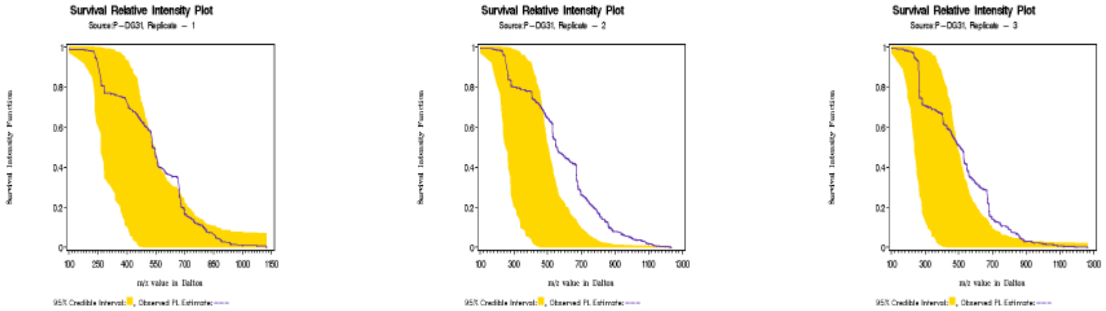

(a) Replicate 1. (b) Replicate 2. (c) Replicate 3.

Figure 2: SRIF plot for patient P-DG31. The large deviation from the 95% confidence band is an indication of irregular metabolic behavior.

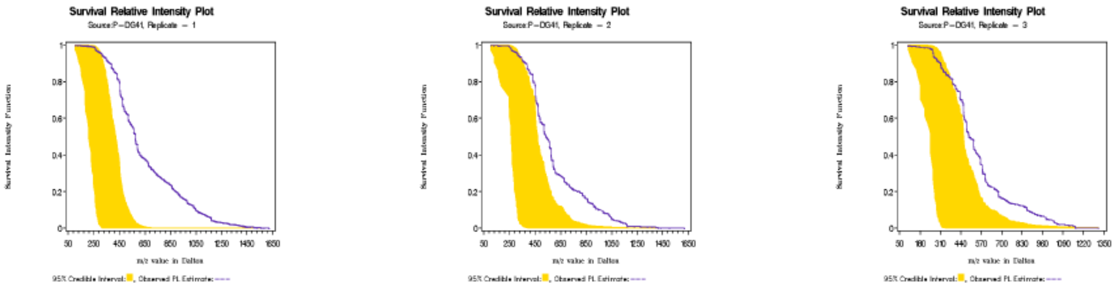

(a) Replicate 1. (b) Replicate 2. (c) Replicate 3.

Figure 3: SRIF plot for patient P-DG41. The large deviation from the 95% confidence band is an indication of irregular metabolic behavior.

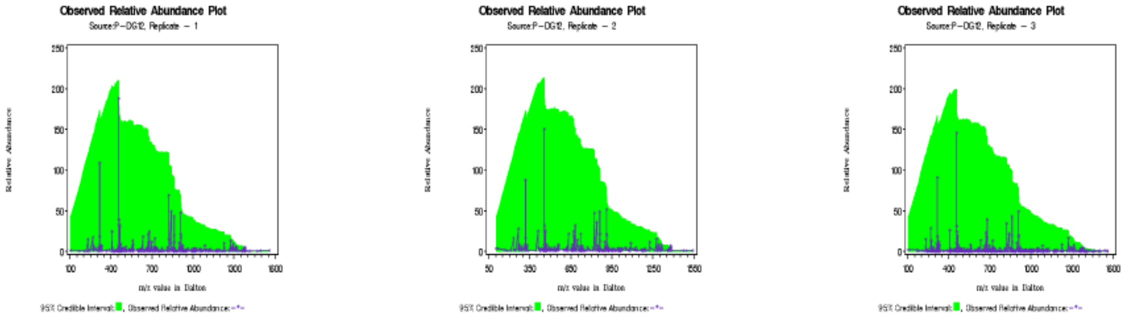

(a) Replicate 1. (b) Replicate 2. (c) Replicate 3.

Figure 4: PRA plot for patient P-DG12. Those m/z values falling outside of 95% credible interval are potential biomarkers.

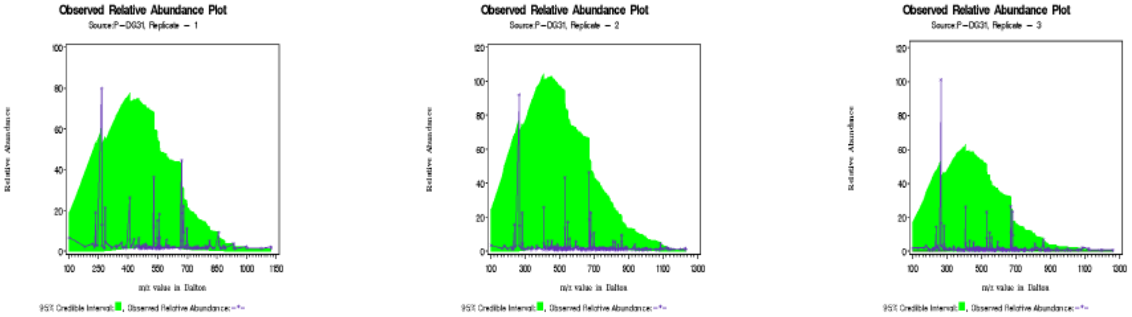

(a) Replicate 1. (b) Replicate 2. (c) Replicate 3.

Figure 5: PRA plot for patient P-DG31. Those m/z values falling outside of 95% credible interval are potential biomarkers.

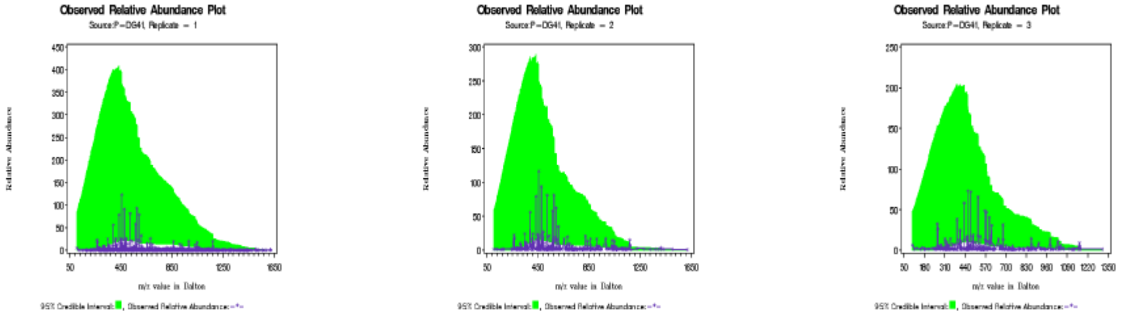

(a) Replicate 1. (b) Replicate 2. (c) Replicate 3.

Figure 6: PRA plot for patient P-DG41. Those m/z values falling outside of 95% credible interval are potential biomarkers.

As discussed in the main paper SRIF and PRA plot has two distinct purposes namely, 1> overall subject diagnostic (more global in m/z scale) 2> potential biomarker identification (more localized). Hence in SRIF a broad picture of subjects well being is captured through the plot at

much higher resolution. For PRA, we concentrate more on the finer scale to determine the list of m/z values falling outside HPD credible interval obtained from the controls. We hypothesized that for subjects with some disorder (trauma here) we will see much outlying m/z value, which is presented at PRA plot for three patients in figure 4-6. This will not be the case for a normal individual (please see figure 5 of the original paper). It is important to note that prior to SRIF and PRA based analysis, all the subject specific medications (for both control and patient) should be eliminated otherwise this may lead us to false discovery of medications as biomarker.

3. Additional Model Comparison Results

Tables 1-3 show different model parameter estimates, standard deviations and their HPD credible intervals. Notably we are not plugging the posterior estimates of different parameters directly during estimation stage. Details of pooling control subjects are being explained latter on. Tables 4-8 represents model comparison results for three models under different model selection criteria. Within the domain of these model choices we exhaustively searched for the best model. An additional flexibility analysis is also reported latter to show that by increasing the number of mixture parameters, we will not gain much in terms capturing local features.

Estimates of the parameters for 12 subjects for Model 1

Model 1: Extreme Value							
Subject ID<br>P -Patient<br>C -Control	Parameter Estimate<br>Posterior mean, S.D. and 95% H.P.D. Credible Interval						
	η_1	η_2	η_3	η_4	η_5	α	β
P-DG1	0.243	0.155	0.179	0.154	0.205	3.065	0.152
	0.059	0.055	0.057	0.059	0.05	0.509	0.156
	(0.127,0.354)	(0.077,0.301)	(0.052,0.304)	(0.07,0.312)	(0.04,0.374)	(2.135,4.144)	(0.11,0.62)
P-DG3	0.239	0.179	0.152	0.155	0.213	3.065	0.154
	0.062	0.057	0.057	0.057	0.075	0.475	0.2
	(0.115,0.354)	(0.063,0.295)	(0.074,0.302)	(0.07,0.293)	(0.06,0.354)	(2.2,4.16)	(0.11,0.59)
P-DG12	0.24	0.152	0.15	0.156	0.211	3.027	0.175
	0.06	0.057	0.055	0.059	0.079	0.45	0.215
	(0.112,0.346)	(0.077,0.303)	(0.079,0.304)	(0.065,0.295)	(0.051,0.373)	(2.19,4.11)	(0.12,0.62)
P-DG31	0.24	0.151	0.179	0.156	0.212	3.012	0.176
	0.062	0.059	0.057	0.059	0.075	0.435	0.204
	(0.119,0.36)	(0.073,0.305)	(0.059,0.254)	(0.052,0.305)	(0.067,0.376)	(2.497,4.09)	(0.13,0.53)
P-DG41	0.242	0.153	0.179	0.156	0.209	3.009	0.191
	0.06	0.057	0.057	0.061	0.079	0.59	0.265
	(0.129,0.36)	(0.05,0.302)	(0.075,0.293)	(0.065,0.309)	(0.069,0.356)	(2.02,4.36)	(0.1,0.71)
P-DG51	0.197	0.197	0.195	0.196	0.21	2.354	0.276
	0.055	0.055	0.053	0.054	0.101	1.51	0.21
	(0.091,0.302)	(0.093,0.307)	(0.101,0.301)	(0.09,0.295)	(0.024,0.402)	(0.02,5.06)	(0.05,0.65)
C-DG62	0.244	0.154	0.153	0.154	0.204	2.9	0.19
	0.06	0.057	0.055	0.06	0.073	0.501	0.209
	(0.124,0.367)	(0.07,0.295)	(0.077,0.305)	(0.073,0.312)	(0.062,0.364)	(1.73,3.55)	(0.09,0.65)
C-DG63	0.244	0.152	0.151	0.153	0.21	3.02	0.176
	0.055	0.055	0.056	0.055	0.076	0.414	0.165
	(0.141,0.365)	(0.057,0.295)	(0.055,0.294)	(0.05,0.309)	(0.055,0.361)	(2.23,3.52)	(0.12,0.54)
C-DG64	0.242	0.152	0.151	0.153	0.211	2.94	0.177
	0.055	0.057	0.059	0.055	0.075	0.35	0.123
	(0.126,0.36)	(0.05,0.301)	(0.065,0.296)	(0.052,0.3)	(0.076,0.367)	(2.43,3.61)	(0.13,0.41)
C-DG66	0.244	0.153	0.151	0.155	0.205	2.906	0.176
	0.055	0.055	0.054	0.059	0.076	0.313	0.133
	(0.127,0.354)	(0.052,0.299)	(0.05,0.292)	(0.075,0.304)	(0.03,0.346)	(2.5,3.62)	(0.14,0.41)
C-DG70	0.245	0.156	0.152	0.154	0.203	2.579	0.152
	0.061	0.06	0.055	0.06	0.079	0.545	0.255
	(0.122,0.36)	(0.069,0.309)	(0.065,0.299)	(0.071,0.31)	(0.056,0.375)	(1.96,4.19)	(0.09,0.67)
C-DG72	0.241	0.151	0.15	0.156	0.212	3.01	0.176
	0.093	0.099	0.095	0.099	0.059	0.51	0.123
	(0.119,0.365)	(0.07,0.305)	(0.065,0.254)	(0.053,0.305)	(0.062,0.376)	(2.494,4.07)	(0.13,0.53)

Table 1

Estimates of the parameters for 12 subjects for Model 2

Model 2: Double Exponential

Subject ID<br>P -Patient<br>C -Control	Parameter Estimate<br>Posterior mean, S.D. and 95% H.P.D. Credible Interval						
	η_1	η_2	η_3	η_4	η_5	α	β
P-DG1	0.139<br>0.14<br>(0,0.23)	0.07<br>0.138<br>(0,0.232)	0.036<br>0.114<br>(0,0.214)	0.019<br>0.09<br>(0,0.198)	0.73<br>0.152<br>(0,0.802)	2.51<br>0.274<br>(1.984,2.94)	0.127<br>0.171<br>(0.068,0.403)
P-DG3	0.199<br>0.081<br>(0,0.23)	0.103<br>0.117<br>(0,0.232)	0.059<br>0.095<br>(0,0.214)	0.044<br>0.086<br>(0,0.198)	0.594<br>0.192<br>(0.128,0.786)	2.65<br>0.193<br>(2.183,2.972)	0.105<br>0.053<br>(0.059,0.232)
P-DG12	0.135<br>0.114<br>(0,0.213)	0.073<br>0.124<br>(0,0.232)	0.052<br>0.134<br>(0,0.269)	0.019<br>0.078<br>(0,0.198)	0.72<br>0.152<br>(0.097,0.803)	2.51<br>0.21<br>(2.103,2.799)	0.109<br>0.083<br>(0.062,0.327)
P-DG31	0.141<br>0.158<br>(0,0.231)	0.065<br>0.127<br>(0,0.232)	0.034<br>0.119<br>(0,0.214)	0.026<br>0.122<br>(0,0.198)	0.732<br>0.165<br>(0,0.802)	2.405<br>0.298<br>(1.93,2.921)	0.129<br>0.111<br>(0.074,0.39)
P-DG41	0.179<br>0.107<br>(0,0.23)	0.093<br>0.119<br>(0,0.232)	0.052<br>0.99<br>(0,0.214)	0.034<br>0.087<br>(0,0.198)	0.641<br>0.181<br>(0.128,0.786)	2.561<br>0.182<br>(2.16,2.78)	0.082<br>0.054<br>(0.04,0.216)
P-DG51	0.157<br>0.154<br>(0,0.23)	0.065<br>0.134<br>(0,0.232)	0.041<br>0.125<br>(0,0.214)	0.027<br>0.106<br>(0,0.198)	0.71<br>0.19<br>(0,0.802)	2.439<br>2.903<br>(-3.3,8.19)	0.75<br>17.371<br>(0.01,25.81)
C-DG62	0.134<br>0.134<br>(0,0.23)	0.071<br>0.141<br>(0,0.232)	0.03<br>0.1<br>(0,0.214)	0.021<br>0.108<br>(0,0.198)	0.741<br>0.147<br>(0,0.803)	2.436<br>0.27<br>(2,2.703)	0.07<br>30.1<br>(0.06,0.33)
C-DG63	0.139<br>0.135<br>(0,0.23)	0.067<br>0.137<br>(0,0.232)	0.042<br>0.125<br>(0,0.215)	0.018<br>0.099<br>(0,0.198)	0.73<br>0.158<br>(0,0.802)	2.464<br>0.273<br>(1.934,2.856)	1.01<br>30.3<br>(0.071,1.41)
C-DG64	0.135<br>0.141<br>(0,0.23)	0.074<br>0.148<br>(0,0.232)	0.037<br>0.123<br>(0,0.215)	0.019<br>0.097<br>(0,0.198)	0.73<br>0.159<br>(0,0.802)	2.38<br>0.299<br>(1.93,2.93)	0.142<br>0.106<br>(0.081,0.474)
C-DG66	0.134<br>0.143<br>(0,0.23)	0.067<br>0.143<br>(0,0.232)	0.036<br>0.119<br>(0,0.214)	0.018<br>0.085<br>(0,0.198)	0.74<br>0.149<br>(0,0.0802)	2.376<br>0.288<br>(1.79,2.79)	0.145<br>0.157<br>(0.081,0.467)
C-DG70	0.143<br>0.143<br>(0,0.23)	0.075<br>0.126<br>(0,0.232)	0.039<br>0.105<br>(0,0.214)	0.019<br>0.091<br>(0,0.198)	0.72<br>0.151<br>(0,0.803)	2.403<br>0.23<br>(2.044,2.676)	0.095<br>0.074<br>(0.054,0.243)
C-DG72	0.142<br>0.144<br>(0,0.23)	0.07<br>0.133<br>(0,0.232)	0.051<br>0.144<br>(0,0.215)	0.024<br>0.104<br>(0,0.198)	0.712<br>0.175<br>(0,0.82)	2.484<br>0.281<br>(2.02,2.97)	0.138<br>0.182<br>(0.072,0.491)

Table 2

Estimates of the parameters for 12 subjects for Model 3

Model 3: Normal							
Subject ID<br>P -Patient<br>C -Control	Parameter Estimate<br>Posterior mean, S.D. and 95% H.P.D. Credible Interval						
	η_1	η_2	η_3	η_4	η_5	α	β
P-DG1	0.309<br>0.079<br>(0.124,0.417)	0.184<br>0.05<br>(0.097,0.292)	0.177<br>0.051<br>(0.084,0.289)	0.179<br>0.049<br>(0.088,0.282)	0.149<br>0.12<br>(0,0.44)	3.356<br>1.787<br>(-1.04,6.698)	34.8<br>74.48<br>(0,39.89)
P-DG3	0.308<br>0.08<br>(0.126,0.42)	0.179<br>0.05<br>(0.081,0.272)	0.181<br>0.05<br>(0.089,0.278)	0.181<br>0.049<br>(0.093,0.281)	0.149<br>0.12<br>(0,0.412)	3.25<br>1.879<br>(-1.496,6.385)	7.236<br>49.255<br>(0,8.072)
P-DG12	0.309<br>0.081<br>(0.121,0.417)	0.179<br>0.049<br>(0.086,0.274)	0.18<br>0.05<br>(0.087,0.277)	0.177<br>0.05<br>(0.092,0.276)	0.152<br>0.124<br>(0,0.427)	3.31<br>1.857<br>(-1.447,6.917)	82.36<br>942.07<br>(0.1,9.747)
P-DG31	0.315<br>0.075<br>(0.133,0.417)	0.184<br>0.05<br>(0.09,0.283)	0.18<br>0.049<br>(0.093,0.284)	0.18<br>0.051<br>(0.085,0.275)	0.139<br>0.113<br>(0,0.406)	3.38<br>1.793<br>(-1.115,6.725)	34.873<br>572.28<br>(0.001,7.635)
P-DG41	0.317<br>0.077<br>(0.137,0.43)	0.184<br>0.05<br>(0.095,0.282)	0.175<br>0.049<br>(0.082,0.267)	0.178<br>0.049<br>(0.091,0.267)	0.145<br>0.117<br>(0,0.416)	3.349<br>1.81<br>(-1.258,6.679)	34.513<br>682.44<br>(0,5.674)
P-DG51	0.197<br>0.055<br>(0.095,0.307)	0.195<br>0.054<br>(0.093,0.304)	0.196<br>0.055<br>(0.1,0.305)	0.2<br>0.056<br>(0.095,0.31)	0.211<br>0.103<br>(0.001,0.356)	2.41<br>2.008<br>(-1.327,6.615)	793.142<br>7232<br>(0.001,75.97)
C-DG62	0.315<br>0.077<br>(0.122,0.419)	0.182<br>0.049<br>(0.089,0.273)	0.18<br>0.051<br>(0.087,0.28)	0.179<br>0.049<br>(0.084,0.265)	0.144<br>0.116<br>(0,0.407)	3.255<br>1.753<br>(-0.64,7.3)	18.208<br>242.2<br>(0,10.39)
C-DG63	0.315<br>0.076<br>(0.133,0.426)	0.179<br>0.05<br>(0.08,0.269)	0.18<br>0.049<br>(0.093,0.288)	0.181<br>0.048<br>(0.092,0.272)	0.142<br>0.114<br>(0,0.402)	3.359<br>1.766<br>(-1.091,6.847)	22.952<br>317.81<br>(0,5.468)
C-DG64	0.315<br>0.075<br>(0.133,0.417)	0.184<br>0.051<br>(0.09,0.283)	0.18<br>0.049<br>(0.093,0.284)	0.18<br>0.051<br>(0.085,0.275)	0.14<br>0.113<br>(0.001,0.406)	3.38<br>1.793<br>(-1.116,6.725)	34.873<br>572.28<br>(0,7.635)
C-DG66	0.316<br>0.076<br>(0.135,0.422)	0.18<br>0.05<br>(0.085,0.275)	0.18<br>0.048<br>(0.083,0.27)	0.18<br>0.049<br>(0.087,0.274)	0.142<br>0.109<br>(0,0.385)	3.3<br>1.694<br>(-0.833,6.675)	54.93<br>154.629<br>(0,11.23)
C-DG70	0.311<br>0.077<br>(0.121,0.411)	0.182<br>0.05<br>(0.083,0.27)	0.18<br>0.05<br>(0.085,0.277)	0.177<br>0.049<br>(0.084,0.267)	0.149<br>0.117<br>(0.001,0.407)	3.19<br>1.783<br>(-0.82,7.05)	66.32<br>545.79<br>(0,7.613)
C-DG72	0.317<br>0.076<br>(0.129,0.425)	0.187<br>0.05<br>(0.086,0.277)	0.178<br>0.049<br>(0.09,0.285)	0.178<br>0.046<br>(0.086,0.261)	0.139<br>0.113<br>(0,0.389)	3.413<br>1.708<br>(-0.523,6.887)	125.34<br>424.95<br>(0,6.949)

Table 3

Log of Pseudo Marginal Likelihood : Model 2 is significantly better than the other two models. However for subject P-DG1 and C-DG72 Model 1 is performing better.

Model 1: Extreme Value, Model 2: Double Exponential, Model 3: Normal

Subject ID P -Patient C -Control	Log of Pseudo Marginal Likelihood			
	Model 1	Model 2	Model 3	Best Model
P-DG1	-11411	-44215	-2.24E+12	Model 1
P-DG3	-91093	-75545	-2.00E+12	Model 2
P-DG12	-17905	-15302	-4.11E+12	Model 2
P-DG31	-9427	-7763	-1.57E+12	Model 2
P-DG41	-27345	-22505	-4.00E+14	Model 2
P-DG51	-63000	-62000	-4.07E+12	Model 2
C-DG62	-11303	-9209	-2.02E+12	Model 2
C-DG63	-13440	-11593	-4.01E+12	Model 2
C-DG64	-11047	-9566	-4.02E+12	Model 2
C-DG66	-7264	-6497	-1.97E+11	Model 2
C-DG70	-15505	-14735	-3.31E+12	Model 2
C-DG72	-20253	-35407	-4.97E+12	Model 1

Table 4

Bayes' Factor : Model 2 is significantly better than the other two models.

Model 1: Extreme Value, Model 2: Double Exponential, Model 3: Normal

Subject ID P -Patient C -Control	Logarithm of Bayes' Factor Obtained via. IWMDE			
	B_{12}	B_{13}	B_{23}	Best Model
P-DG1	-2883.224	319034.218	319034.218	Model 2
P-DG3	-6845.901	377396.254	384242.155	Model 2
P-DG12	-3662.78	12385.733	16048.513	Model 2
P-DG31	-2099.623	238165.032	240264.655	Model 2
P-DG41	-6937.871	230929.214	237867.085	Model 2
P-DG51	1767.618	1052701.959	1050934.341	Model 1
C-DG62	-2804.496	302312.22	305116.716	Model 2
C-DG63	-2094.925	228732.135	230827.06	Model 2
C-DG64	-1696.396	238717.695	240414.091	Model 2
C-DG66	-1263.226	277006.82	278270.046	Model 2
C-DG70	-4477.42	459893.596	464371.016	Model 2
C-DG72	-2929.461	207293.392	210222.853	Model 2

Table 5

Bayesian Information Criterion : Model 2 is significantly better than the other two mo

Model 1: Extreme Value, Model 2: Double Exponential, Model 3: Normal

Subject ID P -Patient C -Control	Bayesian Information Criterion (BIC)			
	Model 1	Model 2	Model 3	Best Model
P-DG1	51015.14	29571.17	126501.2	Model 2
P-DG3	225992.64	191259.5	94067.46	Model 3
P-DG12	45044.66	35004.11	51206.8	Model 2
P-DG31	22695.55	15792	30673.2	Model 2
P-DG41	66365.01	52357.9	65950.52	Model 2
P-DG51	101219.4	97565.3	94144.42	Model 3
C-DG62	25626.55	20119.36	25704	Model 2
C-DG63	30369.75	26365.52	34959.76	Model 2
C-DG64	25551.26	22325.29	31425.52	Model 2
C-DG66	15095.42	15749.02	20624.6	Model 2
C-DG70	46622.19	36429.49	50577.29	Model 2
C-DG72	43376.7	36550.5	47263.23	Model 2

Table 6

Mean Square Error : Model 1 is better for some of the subjects while Model 2 is performing better for some other subjects.

Model 1: Extreme Value, Model 2: Double Exponential, Model 3: Normal

Subject ID P -Patient C -Control	Mean Square Error (MSE)			
	Model 1	Model 2	Model 3	Best Model
P-DG1	2752208.9	66.61	almost ∞	Model 2
P-DG3	170.59	306.46.5	506.76	Model 1
P-DG12	319.84	34.53	1202.7	Model 2
P-DG31	17.7	25.03	373.6	Model 1
P-DG41	58.14	131.5	5980.3	Model 1
P-DG51	9.924E+14	7.084E+11	almost ∞	Model 2
C-DG62	18.23	33.97	7024.6	Model 1
C-DG63	18.84	37.7	3269.6	Model 1
C-DG64	20.68	28.02	1435.5	Model 1
C-DG66	14.6	20.15	20624.6	Model 1
C-DG70	36.32	25.93	574.2	Model 2
C-DG72	51.09	65.84	2.082E+10	Model 1

Table 7

Cross Validation based RMSE : Model 1 is better for some of the subjects while Model 2 is performing better for some other subjects.

Model 1: Extreme Value, Model 2: Double Exponential, Model 3: Normal

Subject ID<br>P -Patient<br>C -Control	Root Mean Square Error (RMSE)			
	Model 1	Model 2	Model 3	Best Model
P-DG1	60882.02	180.329	almost ∞	Model 2
P-DG3	20.85	20.84	631.88	Model 2
P-DG12	22.506	42.55	8002.52	Model 1
P-DG31	71.64	137.95	713.16	Model 1
P-DG41	35.74	87.26	9980.74	Model 1
P-DG51	57.15	57.17	almost ∞	Model 1
C-DG62	7.16	11.03	8025.06	Model 1
C-DG63	38.93	58.07	2286.13	Model 1
C-DG64	33.27	47.62	1435.5	Model 1
C-DG66	45.68	61.07	16624.52	Model 1
C-DG70	16.69	16.67	351.98	Model 2
C-DG72	32.92	10.05	almost ∞	Model 2

Table 8

4. Pooling of Control Subjects to Construct HPD

We are estimating seven parameters namely five mixture parameters $(\boldsymbol{\eta})$, location (α) and scale (β) parameter in $\tilde{H}(t)$ for each subject. These help us estimating relative intensity (hazard) function given by

$$h(t) = \frac{\frac{d}{dt}\tilde{H}(t)\sum_{l=1}^{m}\eta_l Be(\tilde{H}(t); r_l, s_l)}{\{1 - \sum_{l=1}^{m}\eta_l IB(\tilde{H}(t); r_l, s_l)\}^2} .$$

Likelihood for a subject is given in equation (3) of the main paper. Note that for each of the control subjects we are obtaining samples from the posterior distributions of each parameter. Under the hypothesis that control subjects are homogeneous, the posterior parameter distribution should be similar. To illustrate more consider location parameter α only. We obtain posterior samples from all control subjects separately and then mix them together under the hypothesis that posterior distribution of α is same for all controls. Notably this will not be true if underlying hypothesis of homogeneity among controls is violated. Since different trauma types are highly heterogeneous, it is a prime reason that we cannot combine patient samples. Then we repeat the same procedure for other parameters too.

Figure 7 represents SRIF plots for same subject (patient) for two different replicates. Evidently credible intervals are different and apparently may not be clear to the reader why it should be so even for the same patient across replicates. Notably credible intervals are constructed exclusively by combining controls, and of course once posterior samples are obtained they never change. However note that $h(t)$ is also a function of t (proxy of the m/z value) with seven other

parameters and the location of the observed intensities (t) are different even between replicate to replicate. This is depicted in figure 8.

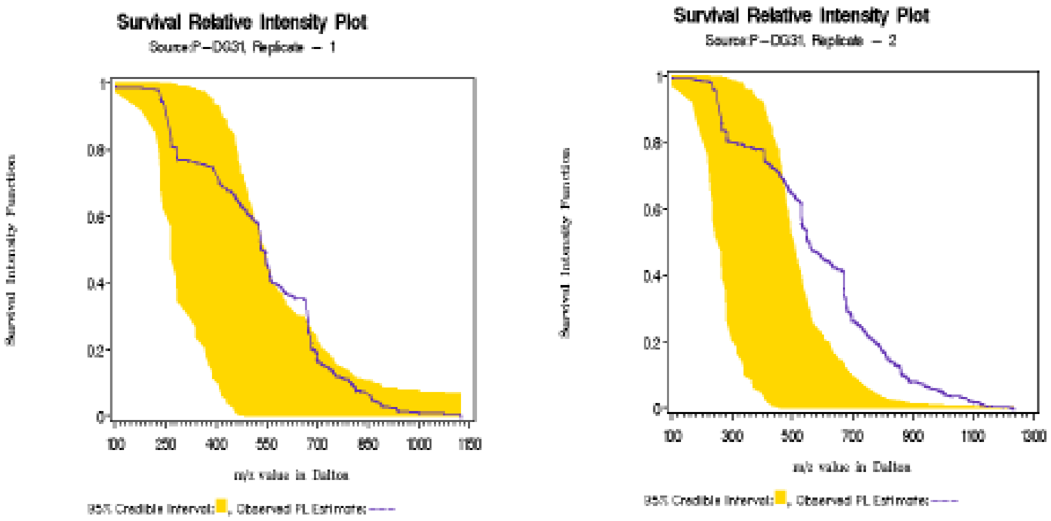

Figure 7

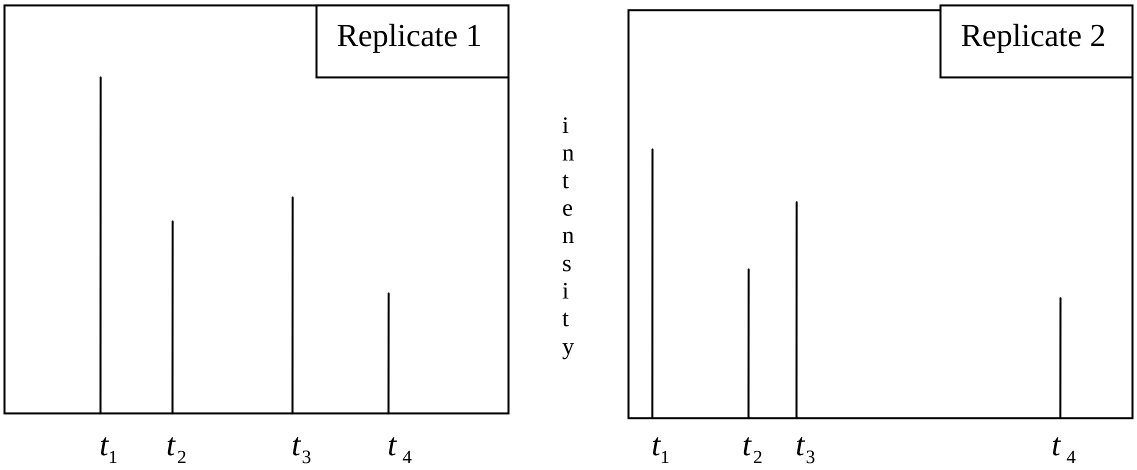

Figure 8

We calculate credible interval for intensity which corresponds to a patient based on a replicate at a position where we observe the intensity value. Since t_1 for replicate 1 is different than t_1 in replicate 2 this implies associated $h(t)$ value and its credible interval will be also different. In fact t_1 denotes the first m/z value (location) of a replicate and t_1 across different replicates has no straight forward relationship except for a place holder. Notably we have not used any alignment algorithm in the current study. However they (t values) will be exactly same if the replicates are in perfect alignment. The PRA estimate is given by

$$\hat{\delta}_t^i = \widehat{h(t)} \times n_t^i \text{ for } t = t_{1_i}, t_{2_i}, \ldots, t_{n_i}.$$

As δ_t is very much depends upon $h(t)$, which in turns depends on observed t of a specific replicate (say i -th), it is necessary to introduce a superscript-i. in δ_t. Since we have many

posterior samples for each parameter from controls, we can obtain several estimate of δ_t for fixed *t* and *i*-th replicate Hence we introduced a Monte Carlo (MC) estimate of δ_t^i as

$$\hat{\delta}_t^i = n_t^i \times \frac{1}{G}\sum_{g=1}^{G}\widehat{h_g(t)}.$$

It is easy to construct HPD credible interval from G-many MC estimates of δ_t^i following Chen-Shao's (JCGS, 1998) algorithm. Notably if there is no variation in the x- axis (observed m/z value) we do not need i-th subscript in δ_t consequently credible intervals will not vary across replicates.

Similar conclusion can be drawn from SRIF which is expressed as

$$\widehat{S_{MC}^i(t)} = \frac{1}{G}\sum_{g=1}^{G}\widehat{S_g^i(t)} = \frac{1}{G}\sum_{g=1}^{G}\prod_{j:t_j\leq t}\left(1-\widehat{h_g(t_j)}\right).$$

Again SRIF depends upon estimation of $h(t)$ and all the above reasoning will also be valid for difference in credible interval across replicates.

5. Some Comments About Flexibility with Five beta Mixtures

A pertinent question is whether the considered model can capture complexity in the data with only five beta mixtures. We performed several flexibility analyses by varying number of mixands at different levels. In our experiment we have tried increasing the number of components from five to ten. While more mixture components will somewhat improve the result however it comes at the expense of increasing the computational cost. We would like to report that for the subject specific global profile estimation (SRIF) five mixands is as good as ten for all three models considered here. However this is highly study specific and we recommended trying several of them in consultation with domain experts. In principle parsimonious model selection should be encouraged. Another way out is to treat the number of mixing components as an unknown quantity which needs to be estimated from the data. However this will come at the increasing cost of computational demand.

Additional References

Geisser, S. (1980). Discussion on sampling and bayes' inference in scientific modeling and robustness (by g.e.p. box). J. R. Statist. Soc. A 143, 416-417.

Gelfand, A. E., Dey, D. K. and Chang, H. (1992). Model determination using predictive distribution with implementation via sampling based methods. In Bernardo, J. M., Berger, J. O., Dawid, A. P. and Smith, A. F. M., editors, Bayesian Statistics 4, pages 147-167. Oxford University Press.

Sinha, D. and Dey, D. K. (1997). Semiparametric bayesian analysis of survival data. Journal of the American Statistical Association 92, 1195-1212.

Chib, S. (1995). Marginal likelihood from the gibbs output. Journal of the American Statistical Association 90, 1313-1321.

Kass, R. and Raftery, A. E. (1995). Bayes factor and model uncertainty. Journal of the American Statistical Association 90, 773-795.

Chen, M. H. (1994). Importnace-weighted marginal bayesian posterior density estimation. Journal of the American Statistical Association 89, 818-824.

Schwartz, G. (1978). Estimating the dimesion of a model. Ann. Statist. 6, 461-466.
